# Early nursing interventions in post-stroke neurological rehabilitation: a comprehensive review of evidence predominantly from high-resource settings

**DOI:** 10.3389/fneur.2026.1920170

**Published:** 2026-09-15

**Authors:** Lina Sun, Lili Xue, Tiantian Ye, Xudong Zhang

**Affiliations:** 1 Encephalopathy Center, Affiliated Hospital of Changchun University of Chinese Medicine, Changchun, Jilin, China; 2 Surgical Comprehensive Department, Erdao Branch, Affiliated Hospital of Changchun University of Chinese Medicine, Changchun, China; 3 The First Department of Cardiology, Affiliated Hospital of Changchun University of Chinese Medicine, Changchun, Jilin, China

**Keywords:** dysphagia, early mobilization, implementation science, neurorehabilitation, nursing interventions, nursing-sensitive outcomes, stroke rehabilitation, stroke-unit care

## Abstract

Stroke remains a leading cause of death and acquired long-term disability worldwide, and the earliest days after onset frequently shape the safety, intensity, continuity, and person-centeredness of subsequent rehabilitation. Early rehabilitation is delivered by interprofessional teams, within which nurses make a distinctive contribution through continuous surveillance, repeated bedside implementation of agreed plans, prevention of avoidable complications, and coordination across shifts and settings. This comprehensive review synthesizes contemporary evidence relevant to early nursing contributions in post-stroke neurological rehabilitation for ischemic and hemorrhagic stroke. Guidelines, systematic reviews, randomized trials, and nursing-science sources were identified through structured searches of MEDLINE, CINAHL, Embase, the Cochrane Library, and named guideline and professional-society resources, then synthesized narratively using a transparent, criteria-based appraisal of evidence strength. Central themes include neurological surveillance, swallow safety, positioning and limb protection, readiness-based mobilization, prevention of immobility-related and iatrogenic complications, communication and cognitive support, mood and fatigue care, dignity-preserving continence rehabilitation, education, secondary prevention, and transitions of care. The strongest evidence supports organized stroke-unit care, timely swallow screening, protocolized management of fever, glycemia, and swallowing, appropriate mechanical venous thromboembolism prevention, and early supported discharge for selected patients. Evidence is more mixed for very early high-dose mobilization, routine splinting, several oral care protocols, and some digital interventions. Most included evidence was generated in high-resource stroke systems, and transferability to lower-resource settings requires contextual adaptation. Early nursing interventions are most effective when their nursing-led and shared components are clearly delineated within standardized yet individualized interprofessional pathways that preserve physiologic stability, dignity, equitable access, and long-term participation.

## Introduction

Stroke is a major global health condition and a central cause of acquired neurological disability, with substantial effects on survival, independence, family functioning, health systems, and social participation (1, 2). Advances in reperfusion therapy, neurocritical care, and organized stroke services have improved acute survival, yet many survivors live with motor impairment, dysphagia, aphasia, cognitive impairment, depression, fatigue, pain, continence disturbance, falls, and restricted participation (3–5). The imperative to rehabilitate begins at admission rather than after medical stabilization is complete. For clarity, this review distinguishes four overlapping phases that are sometimes conflated: hyperacute care (first hours, focused on reperfusion and physiologic stabilization), acute care (approximately days 1–7), early subacute rehabilitation (approximately weeks 1–4), and transition to community (discharge and early follow-up). Early rehabilitation, in this review, refers to goal-directed assessment, safety management, restoration of activity, prevention of complications, and preparation for care transitions delivered during the hyperacute, acute, and early subacute phases following ischemic or hemorrhagic stroke. Nursing-sensitive outcomes are those clinical results, such as aspiration pneumonia, pressure injury, falls, delirium, and catheter-associated infection, that are responsive to the amount or quality of nursing care.

Nurses have a distinctive, but not exclusive, role in this early window because they maintain continuous observation, translate interprofessional plans into repeated daily practice, detect deterioration, prevent avoidable harm, and support patients and caregivers through a period of profound uncertainty (6–9). The nursing contribution extends beyond task completion. It includes neurobehavioral assessment, anticipatory risk management, implementation and reinforcement of agreed positioning and activity plans, structured education, dignity-preserving continence care, medication and risk-factor support, and coordination across settings (10–13).

Throughout this review, the term “nursing intervention” refers to an assessment, preventive action, therapeutic activity, coordination function, or educational practice that nurses lead, deliver, reinforce, monitor, or escalate as part of an interprofessional plan. It does not imply exclusive professional ownership. The distinctive nursing contribution lies in continuous bedside observation, repeated implementation across routine care, timely recognition of change, and feedback to the wider stroke team.

Early nursing practice bridges episodic therapy sessions and the lived reality of recovery during every shift. A patient who practices safe sitting with a therapist may lose benefit if bed positioning, toileting, meals, communication, sleep, and family interaction are not aligned with the same goals. Conversely, nurses who reinforce affected-side awareness, protected arm handling, swallowing precautions, graded transfers, and communication strategies can transform routine care into repeated therapeutic exposure. This continuous integration is especially consequential when therapy time is limited, length of stay is short, or the patient has fluctuating alertness, aphasia, delirium, or fatigue (5, 7, 14).

Despite robust endorsement of organized stroke-unit care, the translation of early rehabilitation principles into consistent bedside practice remains variable. Persistent gaps reflect heterogeneity in stroke severity, staffing constraints, inconsistent protocol fidelity, under-recognition of cognitive and mood needs, inadequate caregiver preparation, and inequitable access to rehabilitation after discharge (15–17). Existing reviews tend to address single domains, such as dysphagia or mobilization, or to list nursing tasks without connecting them to recovery mechanisms, nursing-sensitive outcomes, and implementation. The contribution of this review is not to claim that isolated nursing actions independently cause long-term neurological recovery. Rather, it integrates fragmented evidence across phases of care, differentiates stronger from uncertain practices, and explains how continuous nursing assessment, implementation, reinforcement, coordination, and escalation contribute to the safe and reliable delivery of interprofessional rehabilitation. Figure 1 summarizes the continuum of early nursing priorities across phases of care.

**Figure 1:**
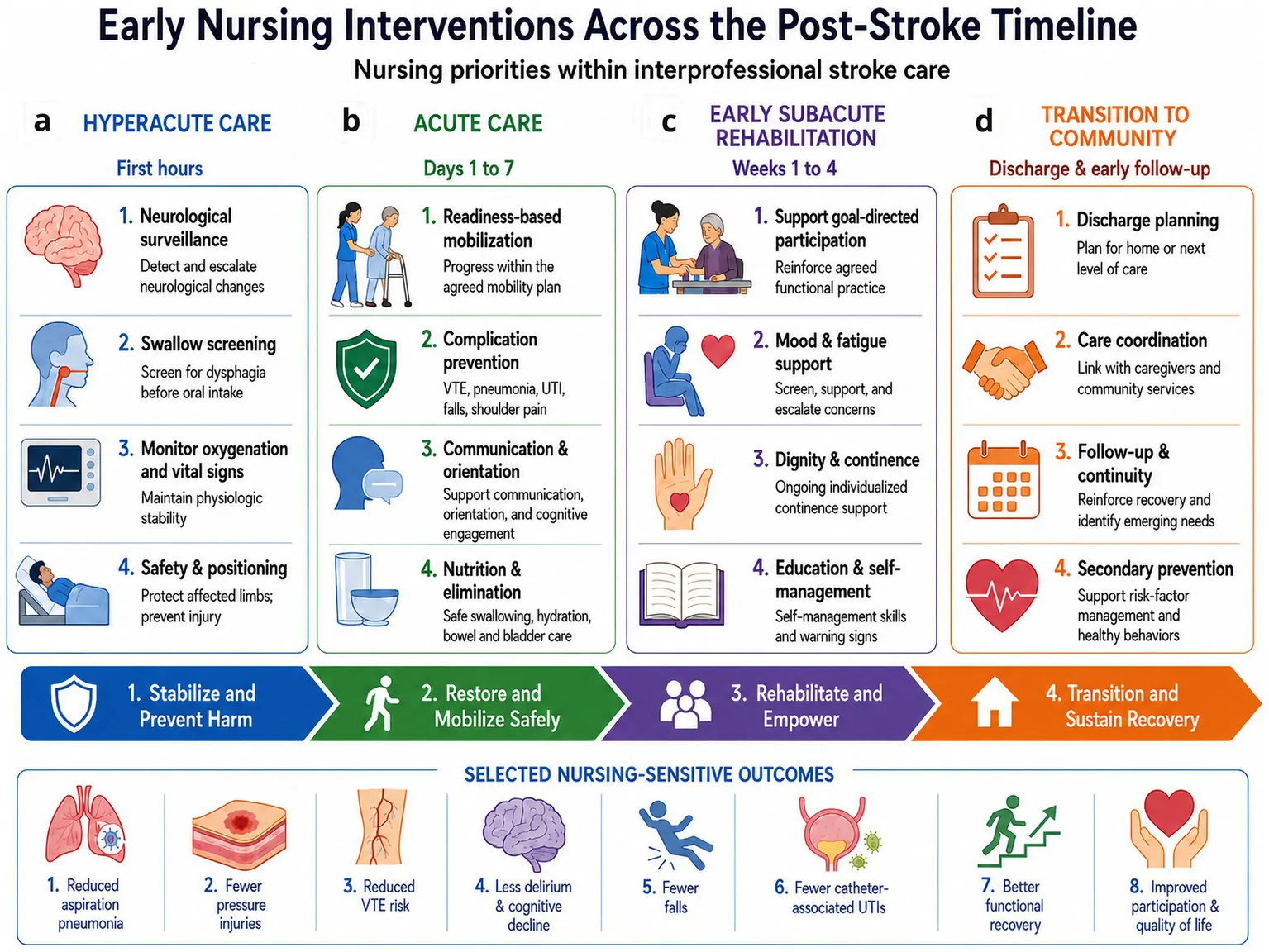
Early nursing interventions across the post-stroke timeline. The simplified figure presents four overlapping phases: hyperacute care, acute care, early subacute rehabilitation, and transition to community. It highlights the principal nursing priorities in each phase and links them to stabilization, safe mobilization, participation, continuity, and selected nursing-sensitive outcomes.

## Evidence identification and synthesis

This article is a comprehensive narrative review rather than a systematic review and is reported in accordance with the Scale for the Assessment of Narrative Review Articles (SANRA) quality criteria (18). The aim was to identify and integrate authoritative, methodologically credible, and clinically relevant evidence on early nursing contributions after stroke, rather than to perform an exhaustive or quantitatively pooled synthesis. The search strategy therefore prioritized current clinical guidelines, systematic reviews, meta-analyses, landmark randomized controlled trials, and high-quality nursing-science sources.

Searches were conducted in MEDLINE (PubMed), CINAHL, Embase, and the Cochrane Library. Targeted website and repository searches covered the American Heart Association and American Stroke Association, European Stroke Organisation, National Institute for Health and Care Excellence, Canadian Stroke Best Practices, the Australian and New Zealand Stroke Foundation Living Clinical Guidelines, the American Association of Neuroscience Nurses, and the Association of Rehabilitation Nurses (19). Search concepts combined population, intervention, setting, and outcome terms, including stroke, cerebrovascular accident, nursing, rehabilitation nursing, acute stroke care, early mobilization, dysphagia, swallow screening, positioning, pressure injury, delirium, continence, communication, secondary prevention, transition of care, and nursing-sensitive outcomes. The search emphasized English-language sources published from 2014 through 2026. Earlier publications were retained when they were foundational randomized trials, remained incorporated into current major guidelines, addressed an intervention for which no newer evidence had superseded the principal finding, or provided an established rehabilitation or implementation framework directly relevant to the review.

Candidate publications identified through database and targeted searches were reviewed by the author team for relevance to adult ischemic or hemorrhagic stroke during the hyperacute, acute, early subacute, or early transition phases. Direct clinical applicability was defined as evidence addressing an assessment, intervention, implementation strategy, or outcome that could reasonably inform bedside nursing practice, interprofessional stroke-unit care, or early post-discharge coordination. Methodological credibility was judged from study design, transparency of methods, consistency with major guidelines, and verifiability of the source. Narrative opinion pieces without an evidentiary basis, predatory publications, and unverifiable sources were excluded. Because this was a narrative synthesis, no formal risk-of-bias scoring, meta-analytic pooling, duplicate systematic screening, or PRISMA flow accounting was performed, and selection necessarily reflects expert judgment. Each major intervention was assigned a strength-of-evidence rating using the explicit scheme in Table 1. This approach distinguishes interventions supported by direct, convergent evidence from those based primarily on consensus or clinical plausibility and signals where causal claims are not warranted.

**Table 1:** Strength-of-evidence rating scheme applied in this review.

Rating	Definition	Typical evidence basis	Interpretation for practice
Strong	Convergent, high-certainty evidence with consistent guideline endorsement	Multiple RCTs or high-quality systematic reviews plus concordant major guidelines	Implement routinely with fidelity
Moderate	Generally consistent evidence with some limitations in quantity, directness, or certainty	Limited RCTs, robust observational data, or systematic reviews with heterogeneity	Implement with individualized judgment
Mixed or limited	Conflicting, sparse, or indirect evidence	Discordant trials, small studies, or evidence extrapolated from related populations	Use selectively; document reasoning
Emerging or consensus	Insufficient trial evidence; supported by expert consensus or clinical plausibility	Guideline consensus statements, mechanistic rationale, or nursing-sensitive plausibility	Reasonable practice pending better evidence

RCT = randomized controlled trial. Ratings reflect the authors’ structured appraisal of guideline endorsement, randomized and systematic-review evidence, consistency, and directness. They are interpretive rather than the product of formal GRADE scoring and are intended to convey relative certainty and discourage causal overstatement.

## Pathophysiological and rehabilitation rationale

Stroke produces focal and network-level disruption through ischemia, hemorrhage, edema, inflammation, diaschisis, impaired perfusion, and secondary injury. Clinical expression may include motor weakness or paresis, sensory loss, neglect, apraxia, dysphagia, aphasia, dysarthria, visual field loss, cognitive impairment, emotional dysregulation, and reduced arousal (3, 20–22). Recovery depends partly on spontaneous biological processes, including resolution of edema and restoration of perfusion, and partly on experience-dependent neuroplasticity shaped by motor relearning, sensory integration, attention, repetition, salience, feedback, and progressive task practice (23–27).

Neuroplasticity is not a purely motor phenomenon. Swallowing recovery, language relearning, attentional control, body schema, emotional regulation, and self-management each require repeated, meaningful practice supported by a safe and enriched care environment. Nurses, physicians, therapists, speech-language pathologists, care assistants, patients, and families collectively shape this environment. Nurses contribute distinctively by sustaining agreed strategies across stimulation, sleep, comfort, nutrition, hydration, communication, family engagement, and routine activities throughout the day and across shifts. Supported conversation can reduce frustration and enable participation in goal setting, while consistent team-based cueing during grooming or toileting can reinforce neglect strategies and promote carryover beyond formal therapy sessions (28–30).

Early nursing contributions within team-based care may influence this biological and behavioral environment, although the causal effect of individual nursing actions is difficult to isolate. Hypoxia, aspiration, fever, hyperglycemia, dehydration, malnutrition, infection, pain, delirium, immobility, shoulder trauma, pressure injury, and sleep disruption are associated with reduced participation and may amplify neurological vulnerability (6, 31–34). Conversely, safe positioning, swallow protection, oral care, early orientation, timely escalation, graded activity exposure, sleep protection, family engagement, and reinforcement of agreed therapy goals are consistent with preserved physiologic stability and greater opportunity for therapeutic practice. Figure 2 presents this proposed pathway as a conceptual model rather than an empirically validated causal chain.

**Figure 2:**
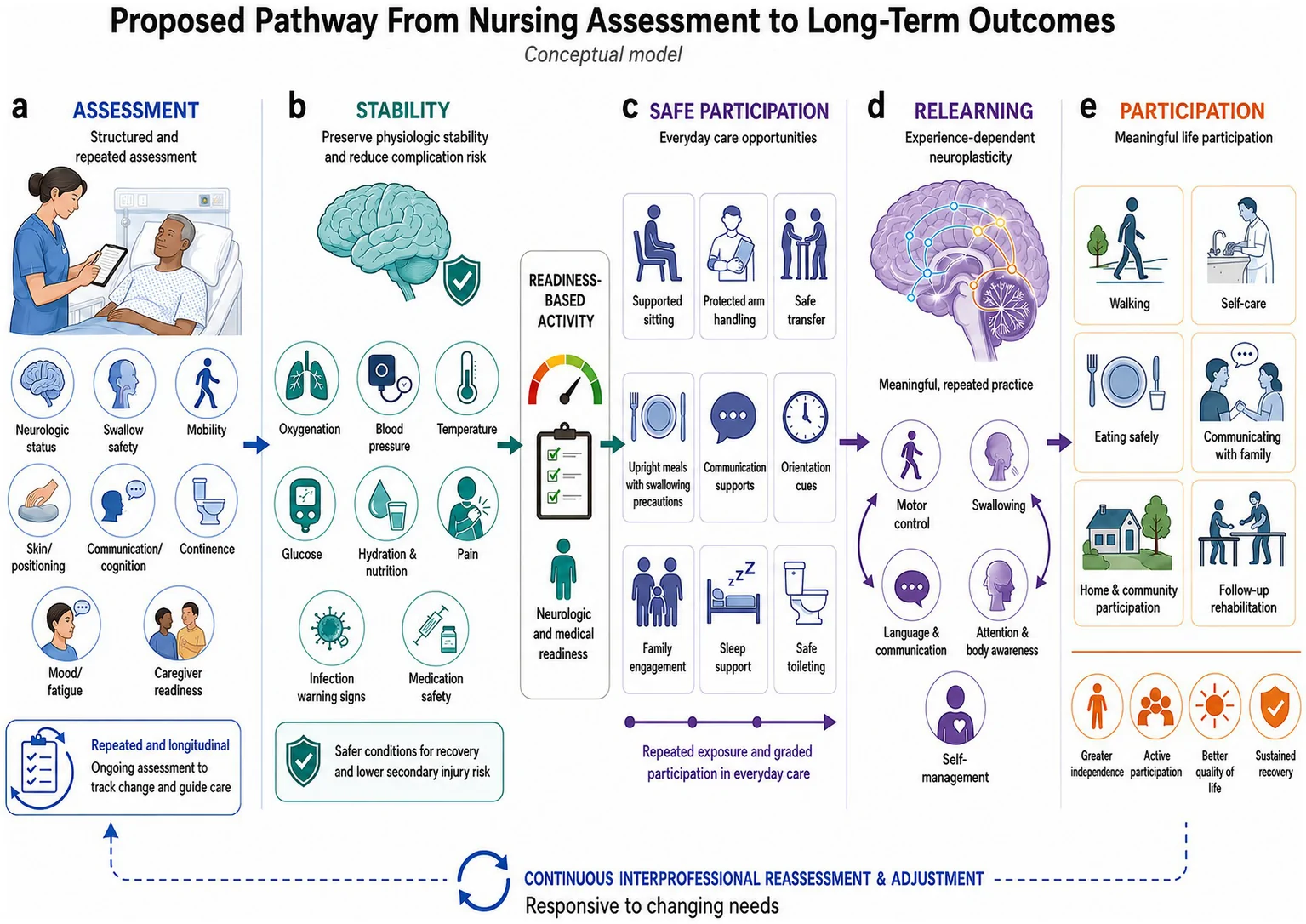
Proposed pathway from nursing assessment to long-term outcomes. This simplified conceptual model, not an empirically validated causal chain, links repeated nursing assessment with physiologic stability, readiness-based activity, safe participation in everyday care, experience-dependent relearning, and meaningful life participation. The feedback loop emphasizes continuous interprofessional reassessment and adjustment.

The timing and dose of early rehabilitation require careful interpretation. Stroke survivors benefit from early organized care and rehabilitation assessment, but very early, high-dose mobilization within the first 24 h was not superior in the AVERT trial and raised concern about excessive intensity in some patients (35–37). Nursing and interprofessional practice should therefore reject the simplistic assumption that earlier and more intensive activity is uniformly better. The goal is readiness-based activation that respects neurological stability, hemodynamics, fatigue, medical restrictions, swallow safety, cognition, and patient priorities (11–13).

## Early nursing assessment

Early nursing assessment is the foundation of safe rehabilitation. It should be structured, repeated, and linked to explicit escalation thresholds. Core neurological elements include level of consciousness, gaze, visual fields, facial movement, limb strength, coordination, sensation, neglect, speech and language, cognition, headache, seizure activity, pain, vital signs, and any change from baseline. The National Institutes of Health Stroke Scale can support standardized neurological communication when nurses are appropriately trained, although it does not replace broader nursing assessment of swallowing, function, skin, continence, mood, fatigue, and caregiver readiness (8, 20, 38, 39).

Assessment must also identify barriers to participation. Dysphagia, aspiration risk, malnutrition, dehydration, aphasia, delirium, depression, anxiety, fatigue, insomnia, urinary incontinence, constipation, immobility, shoulder vulnerability, pressure injury risk, visual or perceptual impairment, falls risk, polypharmacy, limited health literacy, cultural needs, and caregiver burden are all rehabilitation-relevant nursing domains (40–44). Table 2 summarizes practical early assessment domains and their rehabilitation purpose. Assessment findings should lead to a proportionate response, such as urgent medical review for deterioration, referral for specialist swallowing or communication assessment, modification of oral intake or mobility assistance, physical or occupational therapy consultation for function and upper-limb protection, nutrition or psychosocial support, implementation of preventive measures, or tailored teaching for the patient and, with the patient’s agreement, the caregiver.

**Table 2:** Early nursing assessment domains in post-stroke neurological rehabilitation.

Domain	Nursing and shared assessment focus	Clinical purpose	Timing or frequency	Relevant outcomes
Neurological status	Consciousness, NIHSS-informed deficits, pupils, headache, seizures	Detect deterioration and guide escalation	Admission, handoff, status change, protocol intervals	Neurological stability, rescue response
Swallowing and nutrition	Swallow screen, cough, voice, alertness, intake, hydration, weight	Prevent aspiration and malnutrition	Before oral intake and after status change	Pneumonia, hydration, nutrition
Mobility and activity	Sitting balance, transfer level, orthostasis, fatigue, aids	Plan safe activation	Daily and before mobilization	Activity tolerance, falls, independence
Skin and positioning	Pressure risk, limb alignment, edema, shoulder pain	Prevent pressure injury and joint trauma	At admission and each shift	Skin integrity, pain, comfort
Cognition and communication	Aphasia, neglect, delirium, memory, attention, hearing, vision	Enable participation and consent	Daily and after change	Engagement, safety, distress
Mood, sleep, fatigue, pain	Depression, anxiety, sleep, fatigue severity, pain features	Reduce barriers to rehabilitation	Early screening and repeated review	Participation, quality of life
Continence and elimination	Urinary pattern, retention, constipation, catheter need, dignity	Support toileting and skin protection	Admission and daily	Incontinence harms, UTI, dignity
Caregiver and transition	Home context, caregiver capacity, literacy, language, resources	Prepare discharge and continuity	Admission through discharge	Readiness, adherence, access

Note. NIHSS = National Institutes of Health Stroke Scale; UTI = urinary tract infection. Domains reflect guideline, nursing-statement, and interprofessional stroke-care priorities (5–7, 12).

Assessment should be explicitly longitudinal. A patient may be safe to sit at the edge of the bed in the morning but unsafe after a procedure, a sedating medication, fever, hypotension, or a decline in arousal. Likewise, an individual who fails a swallow screen on admission may improve sufficiently to warrant re-evaluation, whereas new aspiration signs may emerge after fatigue or neurological change. Nursing documentation should therefore describe trends, triggers, and functional consequences rather than isolated scores. This trend-based approach improves handoff quality and helps the team distinguish expected recovery, fluctuating attention, medical complications, and neurological worsening (8, 33, 38).

## Nursing contributions to core early rehabilitation interventions

### Neurological monitoring and early detection of deterioration

Continuous nursing surveillance is essential during reperfusion therapy, hemorrhage monitoring, blood pressure management, post-procedure care, and evolving cerebral edema. Nurses identify changes in consciousness, new or worsening weakness, expanding aphasia, severe headache, seizures, aspiration signs, fever, hyperglycemia, hypotension, hypoxia, arrhythmia, bleeding, and access-site complications. Escalation should be protocolized, documented, and communicated with reference to the expected baseline and the exact time of change (3, 6, 8, 39). This is an area of strong, guideline-endorsed evidence. Surveillance supports rehabilitation because deterioration, infection, and physiologic instability delay safe activity and can worsen disability.

Nursing protocols should also guard against quiet deterioration that no single neurological score captures. Reduced oral intake, new incontinence, rising assistance needs, sleep–wake reversal, unexplained agitation, declining sitting tolerance, or caregiver concern may be early signals of infection, delirium, medication adverse effects, hemorrhagic transformation, recurrent stroke, or unmet pain. These observations are often available at the bedside before they surface in formal rounds. A rehabilitation-oriented surveillance culture, therefore, treats bedside concerns as actionable clinical data rather than ancillary notes (7, 33, 34).

### Safe positioning, limb protection, and shoulder care

Positioning is a therapeutic intervention that protects airway alignment, skin, joints, perception, and comfort. All professionals and caregivers involved in positioning, transfers, and personal care should avoid traction on the hemiparetic arm and follow the agreed limb-protection plan. Nurses contribute by applying and reinforcing these precautions during routine care, supporting the shoulder in bed and chair, protecting the hand from edema and trauma, promoting neutral alignment, and reporting changes in pain, tone, edema, or movement to occupational and physical therapists (12, 45, 46). Evidence supporting positioning and shoulder protection rests largely on guideline consensus, and evidence for routine splinting or prolonged stretch is mixed or limited. Positioning should therefore be individualized, reassessed, and integrated with active movement and specialist therapy input (5, 47).

Positioning also serves cognitive and perceptual functions. Bedside layout, approach from the affected side when appropriate, visible clocks and call bells, safe footwear, and placement of personal items can encourage scanning, orientation, and independence. These modifications must be individualized because neglect, visual field loss, impulsivity, sensory loss, and aphasia require different cueing strategies. Nurses can reinforce the team’s plan by encouraging safe participation of the affected limb in bilateral activities while preventing traction injury, pain, and frustration. This balance requires close communication with therapy colleagues and repeated reassessment as tone, edema, and movement change (5, 45).

### Readiness-based mobilization and activity progression

Mobilization encompasses sitting, standing, transfers, walking, task practice, and progressively increasing upright tolerance. Within an interprofessional mobility plan, nurses contribute readiness screening, orthostatic and neurological observation, safe transfer technique, use of prescribed aids, prevention of overexertion, and reinforcement of therapist recommendations during routine care. Stroke-unit care and early rehabilitation assessment have strong evidentiary support, but evidence on mobilization dose and timing is mixed. High-dose mobilization within the first 24 h should be avoided unless clearly appropriate for the individual clinical context (11, 35, 37). Figure 3 presents a practical nursing readiness algorithm; its thresholds are illustrative prompts for clinical judgment rather than validated cutoffs or a substitute for local medical and therapy guidance.

**Figure 3:**
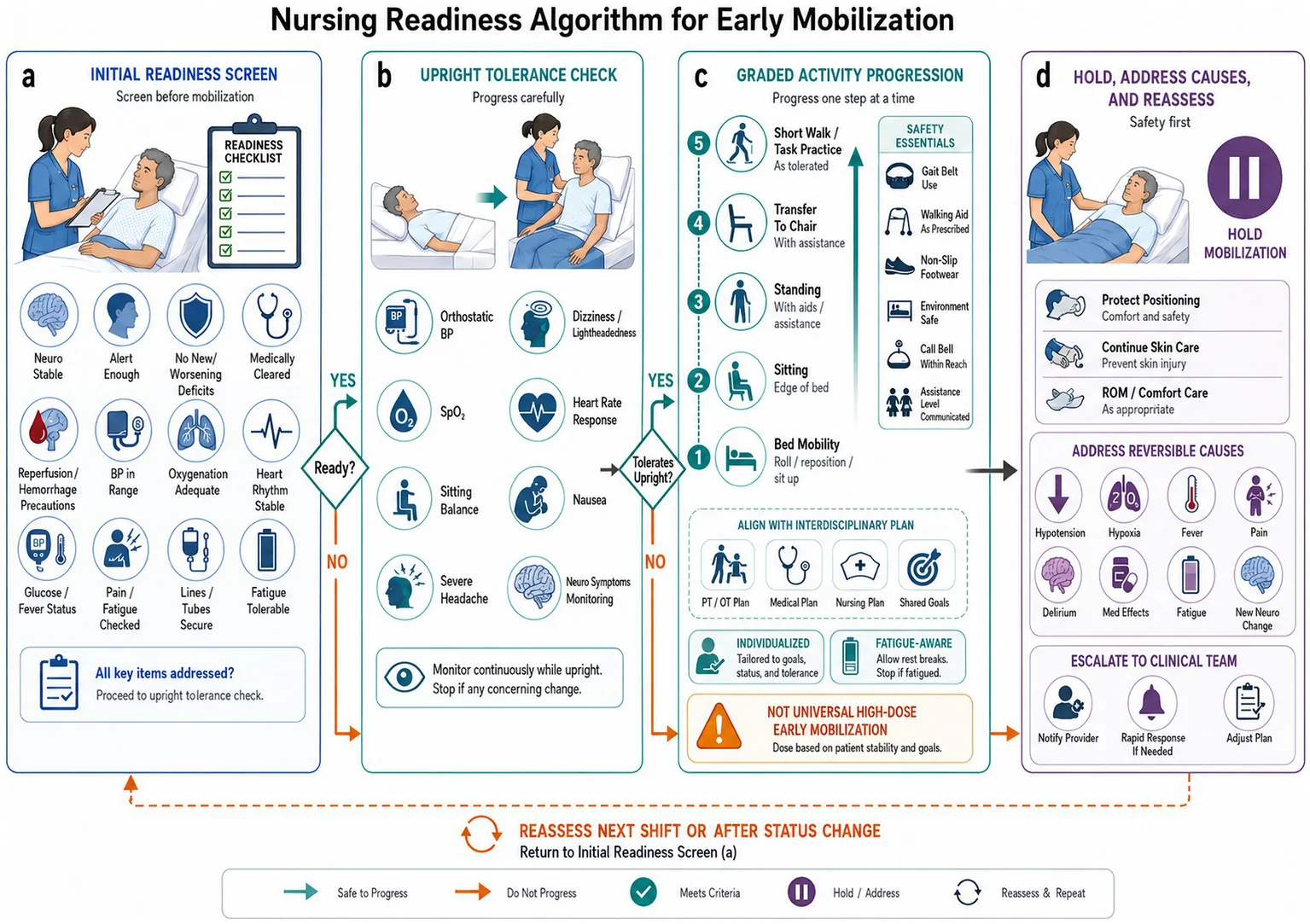
Nursing readiness algorithm for early mobilization. This decision aid guides nurses through an initial readiness screen, upright tolerance check, graded activity progression, and hold, address, and reassess pathway. The algorithm emphasizes neurological stability, alertness, medical clearance, blood pressure, oxygenation, heart rhythm, pain, fatigue, lines and tubes, and tolerance of upright posture. Activity progresses through an agreed interprofessional plan. The thresholds are illustrative prompts for clinical judgment rather than validated cutoffs, and reassessment follows each shift or status change.

### Dysphagia screening, oral care, nutrition, and aspiration prevention

Swallow screening before oral intake is a high-value intervention with strong guideline support and may be undertaken by appropriately trained nurses using a validated local protocol. Patients who fail screening or present with reduced consciousness, wet voice, cough, dysarthria, facial weakness, or recurrent chest symptoms require prompt referral for specialist swallowing assessment and a safe interim plan for nutrition, hydration, and medication administration (7, 41, 45). Oral care, commonly delivered by nurses and care staff, reduces microbial burden and supports comfort, dignity, and readiness for eating, although evidence is moderate and protocols vary across settings (48–51). Nutrition care should address weight, intake, hydration, glycemic management, and dietitian involvement when risk is identified.

Aspiration prevention should not be reduced to diet texture alone. Mealtime care includes alertness checks, upright positioning, pacing, the prescribed level of supervision, oral residue checks, attention to dentures or oral devices, review of medication formulation, and recognition of fatigue. Patients receiving tube feeding still require oral care, aspiration precautions, communication support, and reassessment of swallow potential. Nurses are particularly well placed to observe intake across meals and shifts, identify unintended effects of dysphagia management, such as inadequate fluid intake or medication non-adherence, and communicate these concerns to the speech-language pathologist, dietitian, pharmacist, and medical team (7, 40, 41).

### Prevention of immobility-related and iatrogenic complications

Nursing-sensitive complication prevention spans pneumonia, aspiration, pressure injury, venous thromboembolism, urinary tract infection, falls, delirium, contracture, constipation, dehydration, and shoulder pain. Many preventive actions are shared across professions, while nursing continuity supports their reliable delivery and monitoring. Intermittent pneumatic compression is supported by strong randomized evidence for selected immobile patients without contraindication, whereas routine graduated compression stockings after stroke are not supported by CLOTS trial evidence (3, 52, 53). Pressure injury prevention requires skin inspection, pressure redistribution, moisture management, nutrition support, repositioning, and early mobility, supported by moderate evidence (54, 55). Falls prevention requires individualized mobility assistance, environmental safety, toileting access, appropriate footwear, medication review, and clear communication of assistance levels (56, 57). Delirium prevention through orientation, sleep protection, sensory aids, hydration, infection prevention, and avoidance of unnecessary restraints or catheters rests on emerging and consensus evidence (33, 34). Figure 4 maps complications to corresponding nursing actions within team-based prevention.

**Figure 4:**
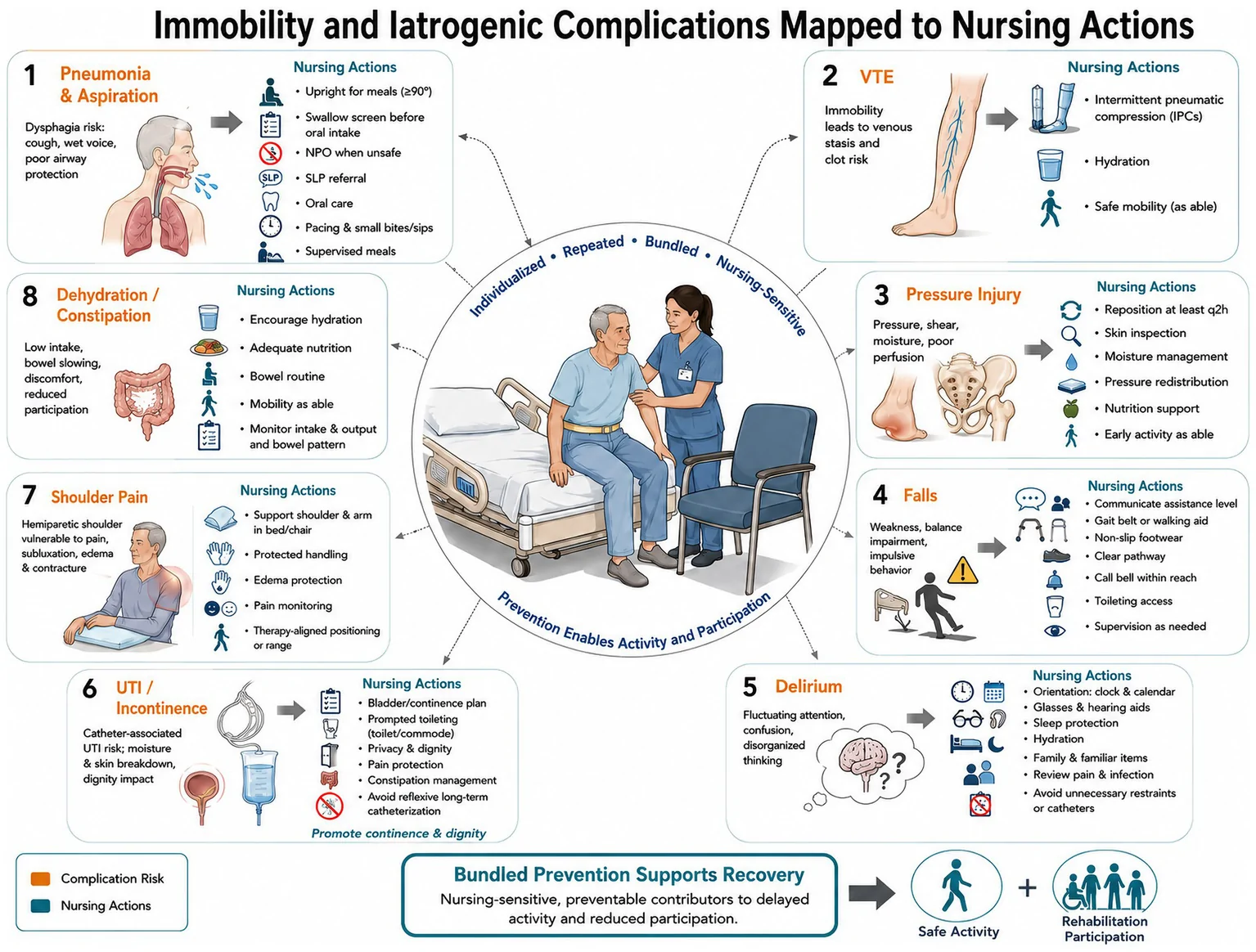
Immobility and iatrogenic complications mapped to nursing actions. The figure maps common complications of immobility and hospital care to practical nursing actions within bundled team-based prevention. The central message is that individualized, repeated prevention supports safe activity and rehabilitation participation.

### Communication support and cognitive orientation

Nurses are often among the first clinicians to observe aphasia, dysarthria, neglect, apraxia, memory deficits, or fluctuating attention during routine care. Formal communication and swallowing assessment remains within the scope of speech-language pathology or another appropriately qualified clinician. Nursing communication support includes short phrases, verification of understanding, writing or picture boards, supported conversation strategies, family involvement, interpreter access, and avoidance of infantilizing language (29, 30). Cognitive orientation can be reinforced through clocks, calendars, predictable sleep–wake routines, glasses and hearing aids, simple choices, familiar objects, and minimization of environmental overload. These measures are supported by moderate and consensus evidence and may improve participation and reduce distress.

Communication access is also a safety intervention. A patient who cannot report pain, dyspnea, urinary urgency, fear, dizziness, or misunderstanding faces increased risk of preventable harm. Following assessment by the speech-language pathologist or other appropriately trained clinician, the agreed communication method should be clearly documented and consistently used by all team members. Nurses reinforce this approach during routine care and report changes in yes-no reliability, comprehension, expression, reading, gesture use, or communication-aid effectiveness. Interpreters should be used for language discordance, and aphasia should never justify exclusion from education, consent, or shared decision-making conversations. Supported communication promotes autonomy and can improve discharge planning (28, 29).

### Mood, motivation, sleep, fatigue, and pain

Depression, anxiety, fatigue, sleep disturbance, and pain may appear early and can substantially reduce engagement with rehabilitation. Nurses should normalize screening, provide steady emotional support, identify suicidal ideation or severe distress, observe temporal patterns and functional effects, and communicate concerning findings to the team. Nurses do not replace specialist diagnosis; persistent or complex symptoms should prompt medical, psychological, neuropsychological, or rehabilitation assessment to distinguish fatigue from depression, apathy, medication effects, sleep disorders, pain, and medical complications (42, 44, 58, 59). Post-stroke depression is associated with poorer functional recovery in observational and systematic-review evidence, although this relationship is correlational and treatment requires individualized evaluation and respect for patient preferences (60–62). Pain assessment should include shoulder pain, central post-stroke pain features, spasticity-related pain, headache, and procedure-related discomfort (63).

### Continence care and dignity-preserving rehabilitation

Urinary incontinence is common after stroke and is associated with skin injury, falls, distress, caregiver burden, and discharge barriers. Nursing care should avoid reflexive long-term catheterization and instead prioritize assessment of type and triggers, toileting assistance, prompted voiding, constipation management, skin protection, privacy, adaptive clothing, mobility aids, and patient goals (64). The evidence base for specific continence interventions after stroke remains mixed or limited and is weaker than for several other domains, but structured assessment and dignity-preserving care are strongly aligned with rehabilitation and person-centered practice and are best regarded as sound clinical practice supported by consensus (45, 65).

### Education, secondary prevention, and transitions of care

Education for stroke survivors and, where appropriate and with the patient’s agreement, family caregivers should begin early and be repeated, multimodal, culturally appropriate, and tailored to cognition, communication, language, health literacy, readiness, and caregiver capacity. Education is a shared interprofessional responsibility. Topics include stroke warning signs, medications, blood pressure, diabetes, lipids, smoking cessation, diet, physical activity, sleep, mood, falls prevention, equipment, appointments, community resources, and realistic recovery expectations (4, 66–69). Caregiver-integrated interventions can improve confidence and continuity when they respect caregiver burden and patient autonomy, with moderate supporting evidence (70, 71). Nurse-led transition and follow-up models show moderate, context-dependent benefits for adherence, early problem identification, and connection to community rehabilitation and should not be overgeneralized across settings (72, 73).

In practice, education should build the patient’s and caregiver’s capability to manage foreseeable situations rather than merely transmit information or restate guidelines. Teach-back and demonstration are preferable to passive instruction. Medication counseling should verify what the patient or caregiver will do when a dose is missed, swallowing is restricted, prescriptions are unaffordable, or adverse effects occur. Transfer and toileting education should include return demonstration, equipment fit, and assessment of environmental barriers. Discharge teaching should clarify who will schedule appointments, monitor symptoms, provide transportation, and contact services if deterioration occurs. This approach treats education as a rehabilitation intervention that builds capability rather than as a discharge formality (70, 73, 74). Table 3 synthesizes major intervention categories, mechanisms, evidence strength, role framing, and cautions. It is a clinical guide rather than a rigid protocol because stroke mechanism, comorbidity, hemorrhage precautions, reperfusion status, cognition, fatigue, goals, and family resources modify priorities.

**Table 3:** Evidence-informed early rehabilitation interventions and nursing contributions.

Intervention category	Nursing contribution within the team	Mechanistic rationale	Evidence strength	Key cautions
Neurological monitoring	Nurse-led standardized surveillance, baseline comparison, timely escalation	Early rescue preserves rehabilitation opportunity	Strong	Avoid vague change descriptions
Swallow safety	Protocol-led nursing screen; safe interim care; SLP referral after failed screen	Reduces aspiration exposure	Strong	Re-screen after status change
Positioning and shoulder care	Apply and reinforce the shared limb-protection plan; monitor pain and edema	Prevents trauma and discomfort	Emerging or consensus	Avoid routine unsupported splinting
Activity progression	Readiness checks and routine-care progression within the agreed mobility plan	Supports task practice and conditioning	Mixed or limited	Avoid excessive very early dose
Oral care and nutrition	Routine oral care and intake monitoring; dietitian and SLP referral when indicated	Reduces microbial load and supports energy	Moderate	Protocol fidelity varies
VTE prevention	Apply and monitor prescribed IPC; support mobility and hydration	Reduces venous stasis	Strong (for IPC)	Avoid routine stockings
Delirium and cognition care	Screen and observe; provide orientation and sleep support; escalate acute change	Improves attention and participation	Emerging or consensus	Do not substitute screening for medical or specialist assessment
Education and transitions	Reinforce shared education, use teach-back, coordinate referrals and follow-up	Improves continuity and self-management	Moderate	Adapt for literacy and language

IPC = intermittent pneumatic compression; NPO = nothing by mouth; SLP = speech-language pathologist; VTE = venous thromboembolism. Evidence strength follows the criteria-based scheme defined in Table 1.

## Interprofessional integration and role delineation

Early stroke rehabilitation is most effective when nurses participate as full members of an interprofessional team rather than as implementers of isolated orders. Role clarity is essential. Physicians lead diagnosis and medical management; physical and occupational therapists assess and prescribe mobility, upper-limb, functional, and equipment strategies; speech-language pathologists lead specialist swallowing and communication assessment; dietitians guide nutrition; pharmacists support medication safety; psychologists and neuropsychologists assess complex mood and cognitive concerns; and social workers address resources and caregiver support. Nurses contribute continuous neurological and functional observation, protocol implementation, reinforcement of agreed strategies during routine care, recognition of change, patient and caregiver education, and coordination across shifts and settings (7, 75, 76).

Nursing continuity adds distinctive value because it connects bedside observation with team decision-making. High-quality handoffs should state neurological baseline, change triggers, swallow status, mobility assistance level, communication method, skin and continence status, delirium risk, therapy goals, family concerns, and discharge barriers. Interprofessional rounds can convert these observations into shared goals such as improved sitting tolerance, safe toileting, progression of oral intake, caregiver transfer training, or prevention of shoulder trauma. Early supported discharge is effective for selected patients when specialist stroke teams, home-based rehabilitation, caregiver preparation, and clear escalation pathways are in place (12, 36, 77).

## Evidence synthesis and critical appraisal

Applying the rating scheme in Table 1, the strongest evidence supports organized stroke-unit care, early interprofessional rehabilitation assessment, swallow screening before oral intake, protocolized management of fever, hyperglycemia, and swallowing, appropriate venous thromboembolism prevention with intermittent pneumatic compression, and early supported discharge for selected patients (31, 32, 36, 53, 75). These are not exclusively nursing interventions. Their reliable delivery depends on coordinated interprofessional systems, within which nursing continuity contributes importantly through repeated assessment, protocol implementation, documentation, escalation, and communication of the patient’s response across shifts.

Moderate, context-dependent evidence supports oral care protocols, caregiver-mediated exercise, structured fatigue management, communication-partner training, and nurse-led secondary prevention or transition support (50, 70, 71, 74). Evidence is mixed or limited for routine splinting, prolonged passive stretching, universal very early intensive mobilization, and several technology-enabled follow-up models (35, 47, 76, 78). In these areas, nurses and the wider team should exercise evidence-informed caution, document individualized reasoning, and seek relevant specialist input.

Critical appraisal is complicated by intervention heterogeneity. Survivors differ by age, lesion type, severity, prestroke disability, cognition, communication, comorbidity, social support, and goals. Care settings differ by nurse staffing, stroke-unit organization, therapy availability, protocols, equipment, and discharge resources. Outcomes also vary, spanning mortality, disability, activity, complications, mood, cognition, quality of life, readmission, caregiver strain, and experience. Table 4 summarizes nursing-sensitive outcomes and quality indicators that can render early rehabilitation nursing more measurable and accountable.

**Table 4:** Nursing-sensitive outcomes and quality indicators.

Outcome domain	Example indicators	Measurement tools	Recommended timing	Relevance to rehabilitation
Safety surveillance	Escalation timeliness, neurological change documentation	NIHSS-informed tools, local rescue metrics	Admission through acute stay	Protects neurological stability
Swallow and aspiration	Screen completion, aspiration events, pneumonia	Validated swallow screen, infection surveillance	Before oral intake and during stay	Enables safe nutrition and therapy
Mobility and function	Transfer assistance level, sitting tolerance, walking readiness	Modified Rankin Scale, Barthel Index, FIM	Daily and discharge	Tracks independence
Skin and positioning	Pressure injury incidence, shoulder pain, edema	Skin tools, pain scales, joint observation	Each shift and discharge	Prevents avoidable disability
Cognition and mood	Delirium episodes, depression, anxiety, fatigue	CAM, PHQ tools, fatigue scales	Early, discharge, follow-up	Improves participation
Continence and dignity	Catheter days, toileting plan, dermatitis	Bladder chart, skin assessment	Daily and discharge	Supports comfort and discharge
Education and transition	Teach-back completion, caregiver competency, referrals	Checklist, documented return demonstration	Before discharge and follow-up	Promotes continuity
Equity	Interpreter use, access barriers, follow-up completion	Equity dashboard, social needs screen	Admission to community	Prevents disparity amplification

CAM = Confusion Assessment Method; FIM = Functional Independence Measure; PHQ = Patient Health Questionnaire. Tools should be selected according to local validation, feasibility, and patient communication needs.

A balanced synthesis must distinguish causality, contribution, and plausibility. Some nursing-delivered or protocol-led actions, such as swallow screening and components of protocolized physiological management, have direct evidence and strong guideline support. Other contributions, such as therapeutic communication, dignity-preserving toileting, family orientation, and reinforcement of therapy goals, are difficult to isolate in trials yet have compelling links to safety, participation, and experience. The absence of isolated trial evidence should not be misread as the absence of value, but it should motivate better measurement, clearer intervention descriptions, and pragmatic designs that capture the real texture of nursing work within interprofessional care (13, 79). This integrative distinction is a principal contribution of the review.

## Implementation considerations

Implementation of the evidence-informed practices summarized in Table 3, including surveillance, swallow safety, complication prevention, readiness-based activity, communication support, education, and transition coordination, requires more than publication of protocols. High-performing units combine stroke-unit organization, adequate staffing, role clarity, competency training, standardized assessments, checklists, electronic health record prompts, escalation pathways, interprofessional rounds, patient and caregiver education materials, audit and feedback, and leadership support (79–83). The Quality in Acute Stroke Care program illustrates how nurse-initiated fever, hyperglycemia, and swallowing protocols can improve adherence and outcomes when implementation is embedded in local workflow and supported by team education and feedback (84–86).

Barriers arise at multiple levels. Patients may experience aphasia, delirium, fatigue, pain, low health literacy, fear of falling, or limited caregiver support. Clinicians may face knowledge gaps, uncertainty about mobilization dose, competing priorities, or inconsistent communication. Units may lack equipment, private space, staffing, specialist therapy, or reliable data systems. Health systems may have fragmented referral pathways, inequitable coverage, rural workforce shortages, and insufficient community rehabilitation capacity (15, 16, 87). Table 5 and Figure 5 translate these barriers into practical, level-specific team strategies and identify the nursing contribution within each strategy.

**Table 5:** Implementation barriers and practical team-based strategies with nursing contributions.

Barrier	Level of care	Practical strategy and nursing contribution	Expected improvement target
Inconsistent assessment	Unit	Standardized admission and shift checklist, with clear escalation responsibilities	Earlier risk recognition
Low protocol fidelity	Clinician	Interprofessional competency training, bedside coaching, and audit-feedback	Reliable swallow and safety practices
Staffing pressure	Organization	Acuity-aware staffing and escalation pathways that protect surveillance and activity	Protected surveillance and mobilization
Fragmented rounds	Team	Shared goals, structured handoff, and nursing feedback from routine care	Aligned rehabilitation plan
Equipment gaps	Unit	Readiness inventory and rapid equipment-access process	Safer transfers and positioning
Language barriers	Patient and family	Interpreter use, accessible materials, and teach-back across the team	Improved understanding
Rural or low-resource access	Health system	Locally adapted referral mapping and follow-up, with cautious use of telehealth	Continuity after discharge
Digital inequity	Health system	Assess device access, connectivity, accessibility, language, and caregiver support	Inclusive remote care

Strategies align with implementation outcome domains of acceptability, adoption, feasibility, fidelity, sustainability, and equity (79). Nursing contributions are presented within interprofessional implementation rather than as exclusive professional ownership.

**Figure 5:**
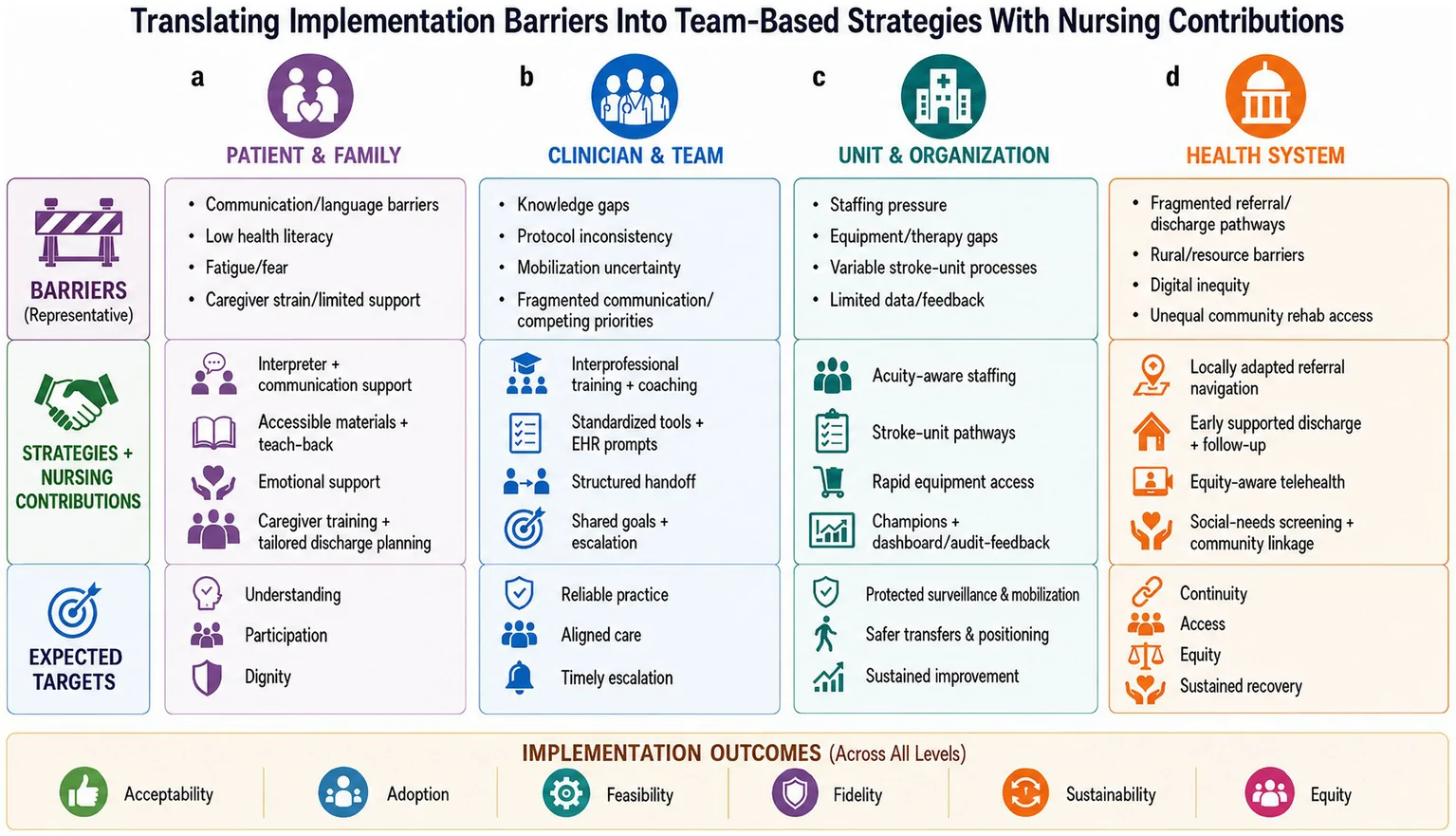
Translating implementation barriers into team-based strategies with nursing contributions. The simplified figure aligns representative barriers, practical strategies, nursing contributions, and expected targets across patient and family, clinician and team, unit and organization, and health-system levels.

### Transferability beyond high-resource stroke systems

Most effectiveness evidence synthesized in this review was generated in high-resource stroke systems. The implications for lower-resource settings should therefore be understood as cautious implementation considerations rather than direct evidence of equivalent effectiveness. Some core practices, including structured observation, swallow safety, oral care, positioning, pressure-injury prevention, dignity-preserving toileting, family education, and safe activity, may be adaptable with low-cost tools when local leadership, training, referral pathways, and workforce roles are considered. Digital tools, remote monitoring, and telerehabilitation may extend continuity, but they can widen inequity through language barriers, poor connectivity, inaccessible interfaces, or absent caregiver support (34, 76, 88–90). Context-specific research led with local stakeholders is required before broad transfer of resource-intensive models.

Sustainability depends on aligning early rehabilitation practices with existing accountability structures. Useful examples include unit dashboards for swallow screening, catheter days, pressure injury, mobilization readiness, delirium screening, patient education, and discharge follow-up; peer champions who provide bedside coaching; and periodic review of cases in which preventable complications delayed therapy or discharge. Such data should drive learning rather than blame. The objective is a high-reliability system in which essential nursing and team-based actions occur consistently despite shift changes, patient complexity, and workload variation (81–83).

## Equity and patient-centered rehabilitation

Equity is a core dimension of early rehabilitation quality. Access to stroke-unit care, rehabilitation intensity, early supported discharge, home therapy, transportation, caregiver training, digital follow-up, and secondary prevention can vary by age, sex, race and ethnicity, rurality, income, insurance, disability, language, cognition, and health literacy (15, 16, 91, 92). Within interprofessional systems, nurses can help reduce inequities by identifying unmet needs early, using interpreters, adapting education, screening for caregiver strain, documenting barriers, and escalating discharge risks. Standardized pathways can also worsen inequities when they ignore culture, communication, finances, inaccessible housing, or caregiver availability.

Patient-centered rehabilitation requires that safety and recovery goals be negotiated rather than imposed. Shared decision-making should inform all preference-sensitive decisions to the extent permitted by clinical urgency, legal requirements, and decision-making capacity. Feeding options, activity goals, discharge plans, assistive devices, medication choices, and caregiver roles are illustrative examples rather than an exhaustive list. Nurses contribute by asking what matters to the patient, identifying valued activities, supporting accessible communication, respecting spiritual and cultural preferences, preserving privacy, and avoiding the assumption that aphasia or cognitive impairment diminishes personhood. Caregiver engagement should include training and emotional support but should not assume unlimited unpaid labor or transfer institutional responsibilities onto families (93, 94).

Equity-sensitive nursing also requires attention to hidden exclusion. Patients with severe aphasia, dementia, homelessness, sensory impairment, undocumented status, or limited family advocacy may be less able to request therapy, understand precautions, or navigate discharge systems. Nurses can counter this by documenting preferences and barriers, requesting interpreters and communication aids, involving social work early, and ensuring that discharge criteria do not privilege patients with greater social resources. Equity metrics should examine who receives education, rehabilitation referrals, follow-up, and complication-prevention interventions, not only aggregate unit outcomes (15, 16).

## Limitations

Several limitations should be considered when interpreting this review. First, as a comprehensive narrative review, it did not use a registered protocol, exhaustive database enumeration, formal risk-of-bias scoring, duplicate systematic screening, or quantitative pooling. Source selection therefore reflects expert judgment and is susceptible to selection and availability bias. The strength-of-evidence ratings are structured and transparent but interpretive and are not equivalent to formal GRADE assessments.

Second, the evidence base itself is heterogeneous. Definitions of early rehabilitation vary across trials and guidelines, mobilization dose and timing are inconsistently reported, and many interventions are studied as bundles, which makes it difficult to isolate the contribution of any single nursing action. Several relevant interventions, including therapeutic communication, dignity-preserving continence care, and family orientation, are intrinsically difficult to randomize and are therefore under-represented in high-certainty evidence.

Third, nursing-specific outcomes are scarce. Many trials report patient-level disability or mortality rather than nursing-sensitive indicators, and follow-up is often short, limiting inference about long-term participation and the durability of early nursing effects. Setting variation, including differences in staffing, stroke-unit organization, therapy access, equipment, and discharge resources, further constrains generalizability, and publication bias may inflate apparent benefits. Most cited evidence derives from high-resource settings, which is now made explicit in the title and scope of the review. Applicability to lower-resource, rural, and culturally diverse contexts should be inferred cautiously rather than assumed. These limitations do not negate the value of integration, but they constrain causal and global claims and reinforce the research priorities outlined below.

## Future directions

Future research should define the active ingredients, timing, dose, and professional contribution of early rehabilitation interventions across stroke severity and care settings. Priorities include pragmatic trials of clearly specified nurse-led and team-based rehabilitation bundles, comparative studies of mobilization dose and readiness criteria, standardized nursing-sensitive outcome sets, continence and dignity interventions, delirium prevention pathways, family-integrated transition models, culturally adapted education, and locally led implementation studies in low-resource and rural settings. Studies should measure fidelity, workload, acceptability, equity, caregiver outcomes, and sustainability, not only patient disability scales (5, 13, 79).

Methodological standards should require precise description of nursing and shared intervention components, professional roles, staffing assumptions, training, duration, frequency, patient eligibility, escalation rules, and adaptations. Trials should capture harms as well as benefits, including falls, fatigue exacerbation, aspiration, delirium, pain, caregiver overload, and inequitable access. Mixed-methods designs are particularly valuable because they can explain why an intervention succeeds in one stroke unit and fails in another. Patient-reported and caregiver-reported outcomes should be collected alongside clinical outcomes so that rehabilitation quality reflects participation, dignity, confidence, and life context (70, 79).

Digital and artificial intelligence tools require rigorous evaluation. Telerehabilitation, nurse-led eHealth follow-up, remote monitoring, and predictive analytics may improve continuity, but their benefits depend on access, usability, clinical integration, and safeguards against algorithmic bias (76, 78, 87). Research should include diverse participants, multilingual materials, caregiver perspectives, and outcomes meaningful to survivors, including participation, dignity, confidence, fatigue, communication, social connection, and return to valued roles.

## Conclusion

Early nursing contributions are foundational to safe, equitable, and effective post-stroke neurological rehabilitation when they are integrated with clearly defined interprofessional roles. Through structured assessment, deterioration detection, protocol implementation, swallow protection, positioning, readiness-based mobilization, complication prevention, communication support, psychosocial screening, continence management, education, secondary prevention, and transition coordination, nurses help create and sustain the conditions in which recovery can occur. The evidence supports organized stroke-unit care and several protocolized interventions, while other areas require individualized team decision-making and stronger nursing-specific research. The significance of early rehabilitation nursing lies not in exclusive ownership of shared interventions, but in continuous observation, repeated implementation, timely escalation, and coordination across everyday care. The central task is not simply to begin rehabilitation early, but to make it safe, meaningful, measurable, equitable, and accessible.
